# Characteristics of environmental RNAi in potato psyllid, *Bactericera cockerelli* (Sulc) (Hemiptera: Psylloidea: Triozidae)

**DOI:** 10.3389/fphys.2022.931951

**Published:** 2022-10-18

**Authors:** Mosharrof Mondal, Megan Carver, Judith K. Brown

**Affiliations:** ^1^ School of Plant Sciences, The University of Arizona, Tucson, AZ, United States; ^2^ RNAissance Ag LLC, St. Louis, MO, United States

**Keywords:** biopesticide, dsRNA, huanglongbing, pest control, RNA-interference

## Abstract

RNA interference (RNAi) has potential to become a major tool for integrated management of insect pests of agricultural crops based on sequence-specificity and low doses of rapidly biodegradable dsRNA. Deploying ‘environmental RNAi’ for control of insect vectors of plant pathogens is of increasing interest for combatting emerging plant diseases. Hemipteran insect vectors, including psyllids, are vascular feeders, making their development difficult to control specifically by targeting with pesticidal chemistries. Psyllids transmit “*Candidatus* Liberibacter solanacearum” the causal organism of potato zebra chip and tomato vein greening diseases, transmitted, respectively, by the potato or tomato psyllid (PoP). Until now, the optimal effective concentration(s) of double-stranded RNA (dsRNA) required for significant gene knockdown and RNAi persistence in PoP have not been determined. The objective of this study was to optimize RNAi in young PoP adults and 3rd instars for screening by oral delivery of dsRNAs. The minimal effective dsRNA concentrations required for robust knockdown and persistence were evaluated by delivering seven concentrations spanning 0.1 ng/μL to 500 ng/μL over post ingestion-access periods (IAP) ranging from 48 h to 12 days. The PoP gene candidates evaluated as targets were vacuolar ATPase subunit A, clathrin heavy chain, and non-fermenting protein 7, which were evaluated for knockdown by qPCR amplification. The minimum and/or the second most effective dsRNA concentration resulting in effective levels of gene knockdown was 100 ng/μL for all three targets. Higher concentrations did not yield further knockdown, indicating potential RISC saturation at the higher doses. Gene silencing post-IAP of 100 ng/μL dsRNA persisted for 3–5 days in adults and nymphs, with the PoP 3rd instar, followed by teneral and mature adults, respectively, exhibiting the most robust RNAi-response.

## Introduction

RNA interference (RNAi) is a biological process that is conserved among eukaryotic organisms. The exploitation of RNAi as a biopesticide technology has potential to expand and possibly reform the repertoire of pesticide regimes available for management of insect pests that damage plants by feeding or serve as vectors of plant pathogens. Though inherently an anti-viral defense system, RNAi also contributes regulatory functions that modulate endogenous gene expression, transcriptionally or post-transcriptionally (Shabalina and Koonin, 2008; Dowling et al., 2017). Small RNAs are the primary effector molecules of the RNAi pathways. These 20–30 nucleotide (nt) long RNAs complementarily bind to the target transcript and trigger degradation of the transcript or blocking of protein synthesis using the transcript as template. Consequently, gene silencing, or RNAi, can be exploited to develop the next generation biopesticides, referred to as ‘environmental RNAi’ (Ivashuta et al., 2015; Mondal et al., 2021). In RNAi technology, long double-stranded RNAs (dsRNA) or single-stranded RNAs (ssRNA) introduced to the target organism are scavenged to the RNAi machinery, usually immediately after ingestion, which is responsible for producing small RNAs that trigger destruction of the target transcript or the inhibition of translation (Agrawal et al., 2003; Baum et al., 2007; Mondal et al., 2020).

The efficacy of RNAi has been shown to result in mortality or other phenotypes similar to that of some traditional chemical pesticides. However, the superior species-specificity and ease of biodegradation of RNAi renders it safer for use in a variety of environments (Christiaens et al., 2022). Also, with respect to human safety, RNAi is considered a superior alternative to chemical pesticides (Baum and Roberts, 2014). Given the desirable features of RNAi, commercial applications that exploit this technology are going to be available for pest management within the next couple of years (Head et al., 2017; Rodrigues et al., 2021). Nonetheless, the success of environmental RNAi biopesticides largely depends on the particular target organism because of variability in responsiveness, or “penetrance”, of the RNAi triggering molecule(s) (Baum and Roberts, 2014). Insects classified in the orders: Blattodea, Coleoptera, and Orthoptera are known to exhibit a robust response, or ‘effective RNAi penetrance’, while dipterans and lepidopterans have been shown to exhibit some extent of recalcitrance to RNAi (Cooper et al., 2019; Zhu and Palli, 2020). Among hemipteran insects, RNAi efficacy has been demonstrated for the genera: *Bemisia*, *Cimex*, *Halyomorpha*, and *Oncopeltus,* whereas many Sternorrhyncha, including aphids and psyllids, exhibit relatively low RNAi penetrance (Moriyama et al., 2016; Ghosh et al., 2017; Mondal et al., 2020; Zhu and Palli, 2020). Several possible biological barriers are recognized that can influence RNAi efficacy among different organisms. Among these are gut pH, RNase activity in the gut, salivary glands, and other organs and tissues, endosomal entrapment of dsRNA, and variable extents of systemic spread of RNAi, once triggered (Parsons et al., 2018; Cooper et al., 2019; Zhu and Palli, 2020).

Biopesticides that exploit RNAi technology are of interest for management of hemipteran pests and vectors of plant viruses, particularly aphids, mealybugs, psyllids, scales, thrips, and whiteflies. The potato or tomato psyllid (PoP), *Bactericera cockerelli* (Sulc, 1909) (Hemiptera: Triozidae) is the insect vector of the causal agent of the zebra chip of potato and tomato vein-greening diseases, which can cause yield loss and reduced tuber and fruit quality, respectively (Insect Pests of Potato, 2013). Both diseases are caused by the psyllid-transmitted fastidious bacterium “*Candidatus*” (*“Ca”*) Liberibacter solanacearum” (CLso) (Brown et al., 2010; Munyaneza, 2012). A distant relative of PoP, the Asian citrus psyllid (ACP) *Diaphorina citri* Kuwayama (Hemiptera: Liviidae), is the insect vector of “*Ca*. Liberibacter asiaticus” (CLas), the causal agent of citrus greening disease, which is recognized as a major threat to the citrus industry worldwide (Halbert and Manjunath, 2004). The internal anatomies of PoP and ACP have been shown to be quite similar (Cicero et al., 2009; Brown, 2016) and render analogous pathophysiological responses to infection by CLso and CLas, respectively (Fisher et al., 2014). Close inspection of psyllid genes involved in the RNAi machinery has revealed similar characteristics of the gene silencing pathways of PoP and ACP, respectively (Fisher et al., 2014; Vyas et al., 2015). Thus, in addition to the development of RNAi biopesticides for management of PoP-transmitted bacterial plant pathogens, PoP has been instrumental in advancing ACP RNAi research. It has been deployed as a surrogate study system for pre-screening dsRNAs in PoP prior to evaluation in the more recalcitrant ACP-CLas pathosystem (Wuriyanghan and Falk, 2013; Fisher et al., 2014). Even so, definitive studies of optimal parameters required for RNAi bioassays in PoP have been quite limited, rendering the relevant characteristics fundamental to effective environmental RNAi in psyllids unknown. Environmental RNAi parameters and paradigms have been established for model organisms, including fruit fly (*Diptera*), red flour beetle (*Lepidoptera)*, and several well-studied non-model insects. However, such parameters may or may not be applicable to evolutionarily distant relatives because of the propensity of different insects to respond variably to RNAi, and because of the fluid nature of RNAi biology itself. Thus, fundamental studies are required to establish optimal RNAi parameters for each insect species of interest. To now, RNAi in psyllids has not been systematically characterized by the evaluation of psyllid-specific dose effects, persistence of gene silencing, or with respect to the developmental stage(s) that are most RNAi-susceptible to the RNAi pathways that can be best exploited for field-ready biopesticide technologies.

Previous RNAi studies involving PoP have used different delivery methods and range of dsRNA concentrations over variable ingestion-access periods (IAP) (Wuriyanghan et al., 2011; Wuriyanghan and Falk, 2013; Galdeano et al., 2017), making comparisons between the different parameters difficult or impossible. To further RNA biopesticide development it has become essential to establish the environmental RNAi characteristics of psyllids, develop standardized approaches for dsRNA target selection, design, and screening, and determine the optimal dsRNA concentration(s) required for optimal knockdown among the different instars. A standardized delivery approach, together with dsRNA reference doses for knocking down candidate gene targets, will enable comparisons between independent laboratories and expedite translation of RNAi to field application for effective psyllid management. Among the most relevant of the unstudied or poorly-studied features of environmental RNAi in PoP and ACP are the optimal knockdown parameters for the different instars, the minimum and maximum effective dsRNA concentration(s) for optimal gene knockdown, and defined points at which maximum silencing can be achieved, or at which silencing reaches a plateau, and at which gene expression returns to baseline after the initial dsRNA-ingestion.

The objective of this study was to determine the optimal parameters for environmental RNAi in psyllids using the PoP-CLso study system and translate the results downstream to the difficult-to-manipulate ACP-CLas study system. In preliminary experiments, ten PoP genes were selected as potential candidates for gene knockdown, identified in a literature survey of model and non-model arthropod targets previously validated by reverse genetics or RNAi knockdown (Royle, 2006; Forgac, 2007; Weiss et al., 2009; Pinheiro et al., 2018), and therefore were considered likely to result in knockdown in psyllids, including PoP. The laboratory PoP transcriptome databases were mined to confirm the presence/expression of orthologous sequences in libraries constructed from adult and immature PoP instars (Fisher et al., 2014). In preliminary experiments, the potential for gene knockdown in psyllids was demonstrated for approximately ten candidate genes, post-dsRNA ingestion by PoP adults and third instar nymphs (0%–40%), adopting the approach that resulted in significant knockdown in adult whiteflies (Vyas et al., 2017).

Based on preliminary results, vacuolar ATPase A subunit (*vATPase-A*), clathrin heavy chain (*CHC*), and non-fermenting protein 7 (*Snf7*) were selected for the study. Gene knockdown was evaluated by quantitative RT-PCR amplification of the respective transcript in PoP adults, post-oral delivery of seven different concentrations of dsRNA, ranging from 0.1 ng/μL to 500 ng/μL. Second, the extent of RNAi persistence in adult PoP was determined for the minimal effective concentration resulting in optimal gene silencing, determined as 100 ng/μL. Finally, to evaluate the possible effects of psyllid developmental stage on PoP RNAi responsiveness (Araujo et al., 2006; Terenius et al., 2011; Ulrich et al., 2015; Vogel et al., 2019; McCusker et al., 2020), RNAi penetrance was evaluated for PoP third instar nymphs, immature or “teneral” adults, and mature or “reproductive stage” adults.

## Materials and methods

### Psyllid colony

The “*Ca’* Liberibacter solanacearum” (CLso)-infected potato psyllid colony was established in 2004 from adult psyllids collected from greenhouse tomato plants in southern Arizona (Brown et al., 2010). The colony has been maintained by serial transfer to 10–12 leaf stage “Roma” tomato plants at approximately every 4–5 wk. Potato psyllids were identified as the “central haplotype” based on genotype-specific SNPs in the cytochrome oxidase I gene (Fisher et al., 2014). Positive infection of the PoP colony by CLso was demonstrated by PCR amplification of the 16S rRNA gene with CLso-specific primers (Liefting et al., 2009), and confirmatory sequencing of amplicons. Ten individual adult psyllids were collected from each colony (cage), year-round for 12 months and assayed for CLso presence to determine percent infectivity. Routinely, the infection rate for the PoP sub-colonies was 90%–100%. The three developmental stages collected from CLso-positive PoP were identified as the 3rd instar (immature), teneral adult, and mature adult by viewing under a dissecting microscope.

### Gene selection for evaluation in the knockdown bioassay

Based on preliminary results (data not shown), the PoP study system was used to evaluate dsRNA-mediated knockdown of PoP vacuolar ATPase A subunit (*vATPase-A*), clathrin heavy chain (*CHC*), and non-fermenting protein 7 (*Snf7*). These targets were selected in part because they were among those exhibiting the highest levels of gene silencing compared to others evaluated in our laboratory. For example, the wings apart-like protein homolog (WAPL), adaptor protein (AP), and Wnt-1 were among the candidates screened that exhibited extremely low to no knockdown (data not shown). Second, the aim was to avoid gene targets for which relative knockdown was less than 40%, to maximize the likelihood that results were reflective of psyllid responsiveness to each increase in dsRNA concentration in the series, for example, 1 ng/μl to 10 ng/μl, and yield a measurable knockdown phenotype for each. Further, the three genes selected are well-known RNAi targets in insects (Baum et al., 2007; Baum and Roberts, 2014; Ulrich et al., 2015; Pereira et al., 2019; Angelotti-Mendonça et al., 2020), and are expressed constitutively in most organisms including insects (Royle, 2006; Forgac, 2007; Weiss et al., 2009; Pinheiro et al., 2018). Finally, expression of each gene has been confirmed by scanning our laboratory transcriptome libraries constructed from PoP adult and nymphal stages, and in the PoP gut and salivary glands of both CLso-infected and -uninfected PoP (data not shown).

### Double-stranded RNA region selection, and *in-vitro* transcription

From the coding region of each gene target, a 200–250 base pair (bp) region was selected as the template for dsRNA synthesis using an *in vitro* transcription reaction. The dsRNA regions were selected bioinformatically using a custom pipeline to ensure that siRNAs produced from the dsRNA target only the gene of interest, e.g., vATPase-A. Briefly, from an initially selected 200–250 bp coding sequence, 21-mers were obtained from the sense and anti-sense strand. Using Bowtie (Langmead et al., 2009), the 21-mers were mapped to the laboratory databases of PoP transcriptome libraries constructed from whole body adults and nymphs and the alimentary canals (guts) and salivary glands (Fisher et al., 2014), allowing one mismatch per read. The number of mapping events against each transcript was counted using BEDtools (Quinlan and Hall, 2010). When a non-target gene was identified by the mapping, a different region of 200–250 bp was selected and the pipeline was re-run. Finally, a region having the minimum predictable off-target matches was selected for each target gene and cloned into the pGEM®-T Easy vector (Promega, Cat. No. A3600) (Data Sheet S2; Supplementary Tables S2–S5). From the psiCHECK-2 vector (Promega, Cat. No. C8021), a 231-nucleotide (nt) region was selected from the firefly luciferase gene sequence available for the firefly *Photinus pyralis* (L.) (Genbank Accession no. AY535007) following pipeline screening as described above, and the PCR amplicons were cloned into the pGEM®-T Easy plasmid vector. The sequence for each amplicon was verified by bi-directional DNA (Sanger) sequencing. The dsRNA was synthesized from the cloned fragment of each target through an *in vitro* transcription reaction using the MEGAscript T7 Transcription Kit (Thermo Fisher Scientific, Cat. No. AM1334), according to the manufacturer’s protocol.

### Potato psyllid ingestion-access to double-stranded RNA

The dsRNAs were delivered to psyllids in a sterile 20% sucrose (w/v) solution in an artificial feeding chamber by pipetting the dsRNA-sucrose solution between two layers of thinly stretched Parafilm M^®^ (Denville Scientific Inc. USA) as previously described in studies of the whitefly (Vyas et al., 2017) and potato psyllid (Paredes-Montero et al., 2022) carried out in this laboratory. The Parafilm M^®^ layers were then attached to a 50 ml polypropylene conical centrifuge tube (Fisher Scientific, catalog # 14-432-22). Ten mL of sterile sucrose solution was prepared for each ingestion-access experiment. The sucrose solution contained one drop (∼50 μL) of green food-coloring (Wilton^®^ Icing Color) used to monitor psyllid ingestion, which was indicated by the green-colored honeydew. Experiments were carried to completion only for those in which psyllids were observed to excrete green honeydew. Three to five independent biological replicates were carried out for each treatment/experiment.

For adult experiments, each replicate consisted of a cohort of ten PoP teneral adults, given a 48-h IAP on 200 μL of sucrose-dsRNA solution. Adult psyllids were collected from an infested, CLso-infected tomato plant using a hand-held aspirator, and transferred to a petri dish containing filter paper for a 4-h starvation period. Psyllids were transferred to the artificial feeding chamber and allowed a 2-day IAP on the dsRNA-containing sucrose solution, after which the dsRNA-sucrose solution was replaced with 20% sucrose. Psyllids were held in the chamber for the duration of the experiment in which dose and RNAi persistence were assessed. Integrity of the dsRNAs were evaluated by agarose gel electrophoresis, post-synthesis, pre-IAP to confirm they were of the expected size, and similarly, post-IAP by electrophoresis of 10 μL volume of the sucrose solution. Only experiments for which dsRNAs were found to be intact on the gel (undegraded), were considered in the analysis reported here. The dsRNA was not replenished or replaced with freshly prepared dsRNA in the “long feeding period” evaluated for RNAi persistence assays.

For immature psyllid experiments, a cohort of fifty 3rd instar nymphs were used in each biological replicate. Third instar nymphs were collected from CLso-infected tomato plants and placed into a (covered) petri dish using a fine paintbrush. Nymphs were starved for 6 h to reduce their vigor and encourage subsequent immediate feeding to discourage their roaming away from the sucrose solution and dying. All live 3rd instar psyllids (at least 30 per replicate) were transferred to 3–4 leaf stage “Roma” tomato seedlings, post-IAP.

### Total RNA isolation, cDNA synthesis, and quantitative RT-PCR

Tubes containing psyllids were submerged in liquid nitrogen for 5 min and transferred to and stored at −80°C. Psyllid cohorts were homogenized in 1 ml Tri Reagent (Zymo Research Cat. No. R2050-1-200) containing 0.5 mm Zirconium beads in a bead-beater (BioSpec Products) (RPI Research Products). The adult and immature psyllids were homogenized in the bead-beater for 8 and 6 min, respectively. Total RNA was isolated using a widely-used Trizol RNA isolation protocol (Rio et al., 2010). Residual DNA was removed from total RNA using the DNA-free™ DNA Removal Kit (Invitrogen, Lot No. 00522653). Using the High-Capacity cDNA Reverse Transcription Kit (Applied Biosystems, Lot No. 00692533), cDNA was synthesized from 2 μg total RNA per sample, according to the manufacturer’s instructions. The qRT-PCR amplification was carried out using a CFX96TM Real-Time PCR System (Bio-Rad Laboratories) and TaqMan RT-PCR master mix (Applied Biosystems; Universal PCR Master Mix, Lot No. 1908161). The psyllid ribosomal protein L5 (RPL5) gene was used as the internal reference gene to normalize candidate gene expression with the ΔΔCt method (Lü et al., 2018). The efficiency of each of the primers/probe combinations was determined based on a 10-fold dilution standard curve (0–10^–6^). Only reaction efficiencies higher than 90% were considered acceptable for qRT-PCR amplification carried out to quantify differential gene expression. The CFX Maestro software v1.1 (Bio-Rad Laboratories) was used to carry out statistical analysis based on the student’s t-test to determine the differences between the means, and Tukey’s HSD was used to identify means that were significantly different. The primers and probe sequences and gene target sequences are provided in the Data Sheet S1.

## Results

### Minimal effective concentration of double-stranded RNA for optimal gene knockdown

**FIGURE 1:**
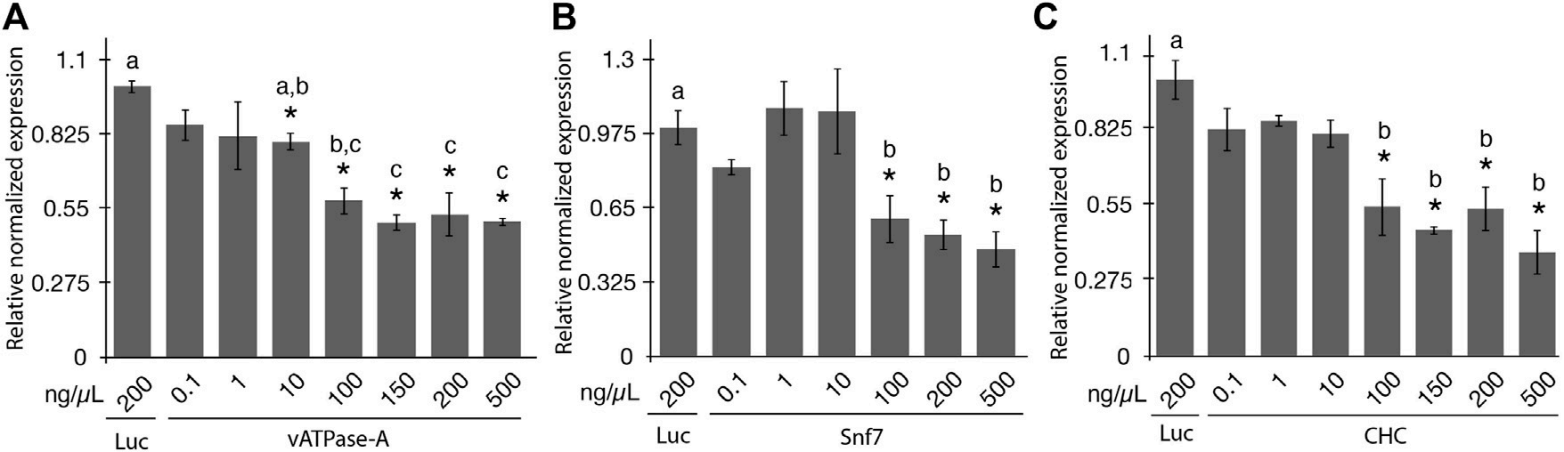
Relative normalized expression of potato psyllid (PoP) genes following ingestion of dsRNAs of the different concentrations **(A–C)**. Relative normalized expression of *vATPase-A* subunit **(A)**, *Snf7* **(B)**, and *CHC* **(C)** genes in “*Candidatus* Liberibacter solanacearum”-infected PoP teneral adults. A 2-day ingestion-access period (IAP) to dsRNAs (100 ng/μL, 200 μL) was provided to psyllids and real time quantitative reverse transcriptase-polymerase chain reaction amplification was carried out 6 days post-IAP. Expression was normalized to the psyllid cohorts receiving 200 ng/μL of the dsRNA luciferase dsRNA non-target control. The Student’s t-test was used to carry out the statistical analysis. Significant groups (*p*-value <0.0.5) are indicated by an asterisk (*) when the results for the experimental and non-target control groups differed. Lower case letters a-c indicate significantly different groups, determined by Tukey’s HSD test (*p*-value 0.05). Each experiment consisted of three to five independent biological replicates, with 10 PoP teneral adults per experiment.

The aim of the study was to evaluate the effectiveness of gene knockdown in the potato psyllid using a range of concentrations of orally-administered synthetic dsRNA using a 48-h post ingestion-access period (IAP). Seven dsRNA concentrations of 0.1, 1, 10, 100, 150, 200, and 500 ng/μL were evaluated for vATPase-A and CHC, and six concentrations were evaluated for Snf7 (all concentrations mentioned above except the 150 ng/μL concentration). The concentrations of dsRNA were selected based on the range of previously reported doses of dsRNA capable of eliciting an RNAi response in PoP or ACP of 0.3 ng/μL to 1,000 ng/μL (Wuriyanghan et al., 2011; El-Shesheny et al., 2013; Wuriyanghan and Falk, 2013; Andrade and Hunter, 2017; Galdeano et al., 2017; Ghosh et al., 2017; Kishk et al., 2017; Yu et al., 2022). Teneral adults were allowed a 2-day post-IAP on 200 μL sterile 20% sucrose solution containing synthetic dsRNA, sandwiched between two layers of parafilm stretched across an artificial feeding chamber, as described previously (Vyas et al., 2017; Paredes-Montero et al., 2022). Psyllids have been reported to show lower sensitivity to RNAi compared to several insects in certain other insect species or orders (Wuriyanghan and Falk, 2013). However, information regarding potential saturation of the RNA-induced silencing complex (RISC) is lacking. Based on the results of a preliminary study conducted in our laboratory (data not shown), a 48-h IAP was selected for the experiments reported here. Gene knockdown was quantified for surviving PoP adults 4 days post-IAP by RT-qPCR amplification. Gene expression was normalized to gene knockdown levels determined by qPCR amplification for the non-target luciferase dsRNA control at 200 ng/μL (Figure 1).

Significant knockdown of *vATPase-A* gene expression was not observed for the dsRNA concentrations of 0.1 and 1 ng/μL. However, increasing the concentration to 10 ng/μL and greater yielded significant gene knockdown. Even so, at a dose of 10 ng/μL only 20% knockdown was observed. Concentrations ranging from 100 ng/μL to 500 ng/μL yielded 42%–50% knockdown, with insignificant variation among the four concentrations (Tukey’s HSD test). Results indicated that the minimal concentration of dsRNA required for optimal knockdown of *vATPase-A* gene was 100 ng/μL. Increasing the concentration to 500 ng/μL dsRNA did not appreciably increase knockdown, which is potentially suggestive of RISC saturation. Although there is no direct evidence for RISC saturation resulting from treatment with high concentrations of siRNA/dsRNA from any insect studies, the phenomenon is well established in mammalian systems (Grimm et al., 2006; Tan et al., 2012; Varley and Desaulniers, 2021). The results of the knockdown experiments for Snf7 and CHC indicated a similar trend with the 100 ng/μL to 500 ng/μL dsRNA treatments resulting in respective gene silencing levels like those observed for the *vATPase-A* gene. These results indicated that RNAi penetrance is lower for psyllids than the whitefly *B. tabaci* and for most coleopteran insects (Mondal et al., 2020; Mehlhorn et al., 2021). Further, a five-fold increase in dsRNA concentration from 100 ng/μL and greater did not result in increased gene knockdown.

### Persistence of RNA interference-induced gene silencing in potato psyllids

**FIGURE 2:**
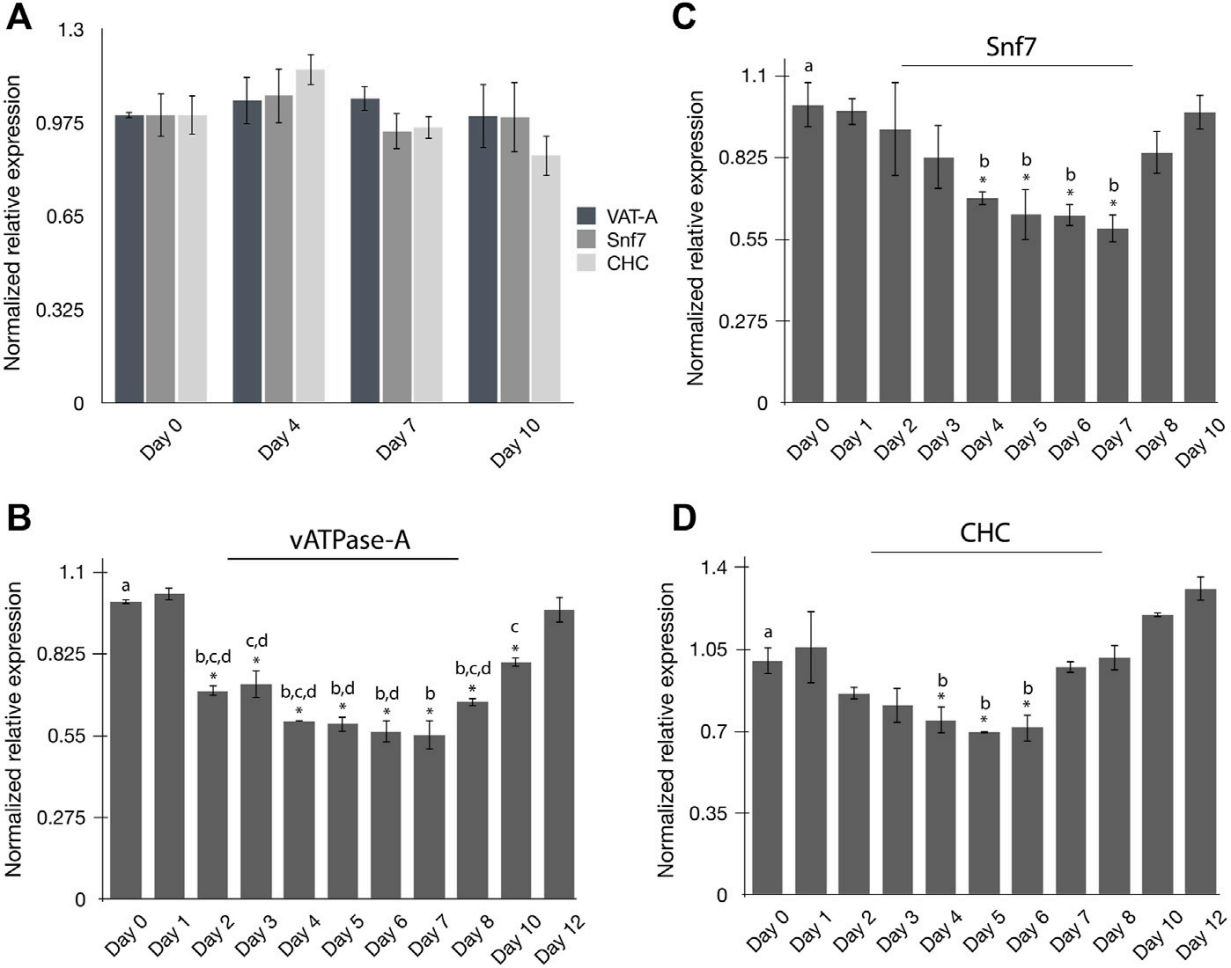
Persistence of RNAi mediated gene silencing in potato psyllids **(A–D)**. **(A)** Endogenous expression of “*Candidatus* Liberibacter solanacearum”-infected PoP *vATPase-A*, *Snf,* and *CHC* genes at day 0, 4, 7 and 10; **(B–D)** Gene expression for candidate genes was normalized to the respective expression level corresponding to that on day zero (0). Time-course study of relative normalized expression of *vATPase* subunit-A **(B)**, *Snf7* **(C)**, and *CHC* **(D)** genes for CLso-infected PoP teneral adults 10-day post-IAP. On day zero (0), psyllids were given an ingestion-access period (IAP) on dsRNA for each respective gene target or the luciferase non-target control (Luc) at 100 ng/μL, 200 μL. On day two (48-h), the dsRNA-sucrose solution was replaced with 20% (w/v) sucrose solution (dsRNA-free). Gene expression was determined by real-time, quantitative reverse transcriptase-polymerase chain reaction amplification, and the results were compared to gene expression levels corresponding to day 0. The asterisk (*) indicates differences in gene expression between day 0 and each respective day for which expression was analyzed, based on the Student’s t-test (*p*-value <0.0.5). By convention, Tukey’s HSD test was applied to groups exhibiting significant differences relative to day 0 expression. The lower case letters a-d indicate different significant groups determined by Tukey’s HSD test (*p*-value 0.05). Each experiment consisted of three to five independent biological replicates for each “dsRNA ingestion”- group consisting of 10 PoP teneral adults per experiment.

The persistence of RNAi-induced gene silencing in psyllids has not been well studied. The experiments were carried out to evaluate the persistence of gene silencing in PoP at the pre-determined minimal effective dsRNA concentration of 100 ng/μL (see Results, above). To this end, the endogenous expression of the three selected target genes, *vATPase-A* (VAT-A), *Snf7*, and *CHC,* was quantified in time-course experiments. On day 0, newly emerged teneral adults were collected from PoP colonies and gene expression was quantified on days 0, 4, 7 and 10 by qRT-PCR amplification (Figure 2A). For all experiments, psyllids were held in an artificial feeding chamber for the duration of the study, as indicated. Methods were as described above for dsRNA ingestion-access bioassays. Relatively consistent gene expression levels, based on knockdown, were observed for the three genes evaluated for both the PoP teneral and mature adult stage psyllids (Figure 2A).

Next, psyllids were allowed a 48-h IAP to the dsRNA in a 20% sucrose (w/v) solution, designating day 0 as the initial or ‘first day’ of dsRNA ingestion-access. Forty-eight hr post-IAP (day 2), the dsRNA-sucrose solution was replaced with 20% (w/v) sucrose-only solution. Gene knockdown was determined by qRT-PCR amplification over the 12-day time course for PoP teneral adults using qRT-PCR. Gene expression was monitored on days 1, 2, 3, 4, 5, 6, 7, 8, 10, and 12 and compared to that for day 0 for *vATPase-A* and *Chc,* while knockdown of the *Snf7* gene expression was measured up to day 10. Knockdown among the three genes was variable, as might be expected with respect to initiating significant level knockdown compared to the day 0 starting point, as was the persistence of silencing (Figure 2). Knockdown of the *vATPase-A* gene expression was significant after 48-h (day 2), and persisted until day10 (8 days post-IAP), with maximum knockdown documented during days 4–7 (Figure 2B). Interestingly, silencing began to decline by day 8 and by day 12 (end of experiment), knockdown of *vATPase-A* was not significant. In contrast, significant knockdown of *Snf7* gene expression was observed on days 4–7, with no significant knockdown detectable by 6–8 days (Figure 2C). The patterns observed for *CHC* and *Snf7* gene knockdown were similar, with significant gene knockdown occurring on days 4–6 and exhibiting a gradual decline to the day 0 levels (Figure 2D). Thus, dsRNA-induced gene silencing was diminished entirely by 4–5 days, post-IAP.

### Comparison of RNAi efficiency for different psyllid developmental stages

**FIGURE 3:**
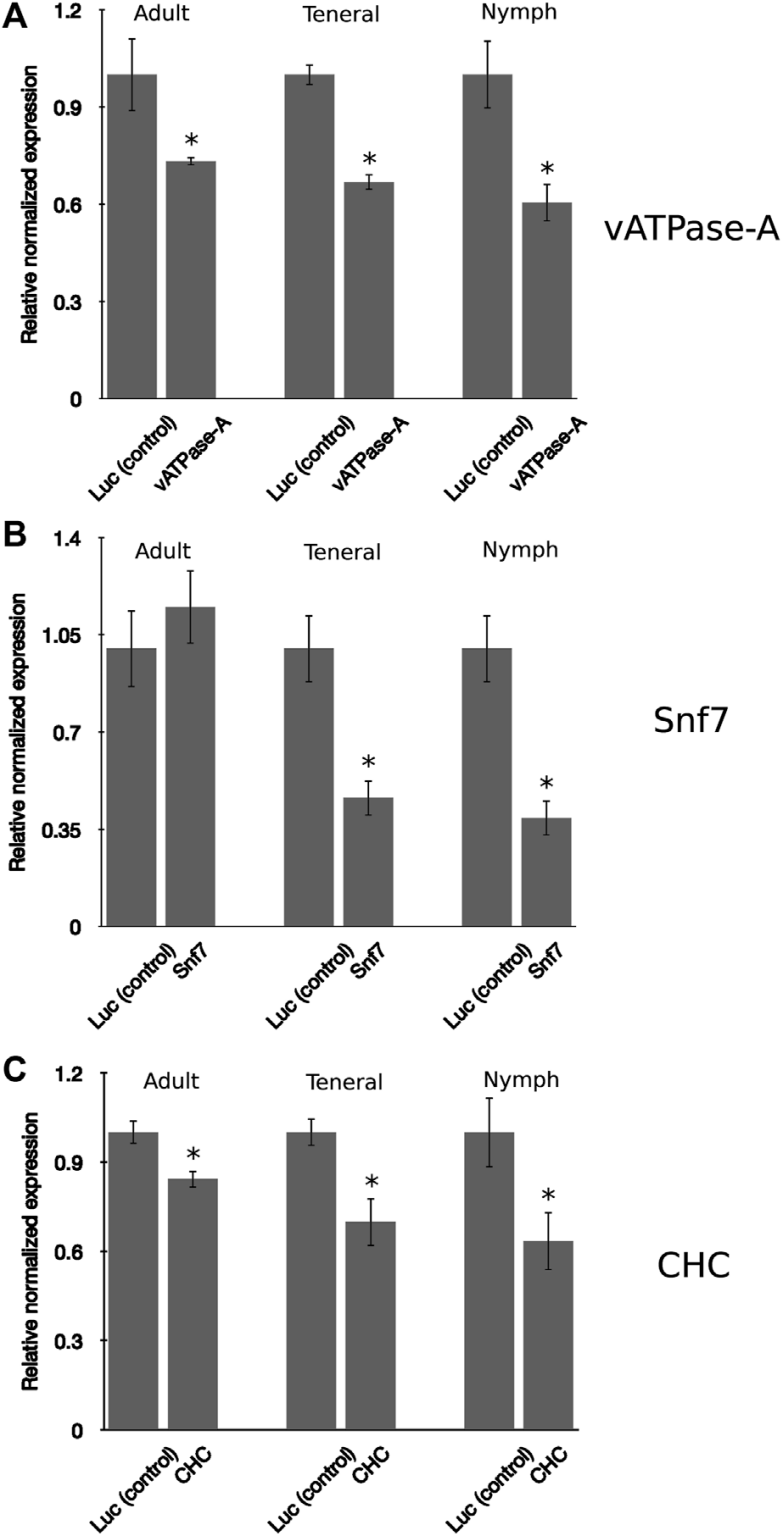
Comparison of gene knockdown levels across the developmental stages in potato psyllid **(A–C)**. Relative normalized expression of *vATPase-A* subunit **(A)**, *Snf7* **(B)**, and *CHC* **(C)** genes in “*Candidatus* Liberibacter solanacearum”-infected mature adults, teneral adults, and 3^rd^ instar nymphs. Psyllids were provided a 2-day inoculation-access period (IAP) on dsRNA for each respective gene target or the luciferase non-target control (Luc) at 100 ng/μL, 200 μL. Gene expression was determined by real time, quantitative reverse-transcriptase polymerase chain reaction amplification, 6 days post-IAP. Relative gene expression was calculated by comparing expression for dsRNA-treated psyllids of each analogous instar cohort, post-IAP on dsRNA for the gene target and non-target Luc control. An asterisk (*) denotes the results of the Student’s t-test (*p*-value <0.0.5) for experimental and non-target control group comparisons. Each experiment consisted of three biological replicates per instar cohort. Eight to ten mature and teneral adults and fifteen to 20 3rd instar nymphs, respectively, were collected 4-days post-IAP for gene expression analysis.

In insects, the developmental stage has been found to influence RNAi penetrance, a phenomenon resulting from differences in the response to exogenous RNAi triggers (Schmitt-Engel et al., 2015). In general, younger insect developmental stages have been found to be more receptive, or sensitive, to knockdown, compared to more advanced life stages (instars) (Guo et al., 2021; Salvador et al., 2021; Willow and Veromann, 2021). The psyllid life cycle consists of five nymphal instar stages, the newly emerged adult, also referred to as a teneral adult, and the mature adult (Butler and Trumble, 2012). In this study, the responses to environmental RNAi were compared for PoP 3rd instar nymphs, teneral adults (light brown or yellow body color), and mature adults (dark brown body color). Psyllids were allowed an IAP on dsRNA solution, v-ATPase-A, Snf7, or CHC at 100 ng/μL, in 20% (w/v) sucrose solution for 48 h. After the 48-h IAP, psyllids were transferred to a 3–4 leaf stage “Roma” tomato seedling. A similar pattern of gene knockdown was observed for all three psyllid life stages, with the nymphal stage exhibiting the greatest sensitivity to environmental RNAi compared to the teneral and mature adult stages, indicating at least some difference in responsiveness by nymphs and adults (Figure 3). A similar trend was observed for the three candidate gene targets, except for Snf7, for which knockdown in adult psyllids was not significantly different from the non-target control, even though significant knockdown was observed for the 3rd instar nymphs and teneral adults (Figure 3B). Knockdown of *vATPase-A* and *CHC* targets for teneral adults was not significantly different from that observed for mature adults. These observations indicate that RNAi penetrance in the 3rd instar psyllid nymphs was significantly higher than for the two older (adult) life stages, indicating that among the developmental stages (instars) examined in this study, potato psyllid nymphs were the most sensitive to environmental RNAi-mediated gene knockdown, compared to the teneral and mature adults.

## Discussion

Three key features of environmental RNAi were characterized for the potato psyllid based on ingestion-access bioassay method in this study. Several other delivery approaches have been evaluated for dsRNA in RNAi bioassays, including injection into the abdomen (Dzitoyeva et al., 2001; Ulrich et al., 2015), topical application to external body parts or imbibition of topically-applied dsRNA (Killiny and Kishk, 2017; Vaschetto, 2022, pp. 253–277), ingestion-access to dsRNA applied to leaf disks (Ghosh et al., 2017), transgenic plant-mediated expression to facilitate phloem delivery with “continuous” exposure by over-expression of dsRNA hairpins (Eakteiman et al., 2018), stem cutting- or root- uptake of dsRNA (Ghosh et al., 2017; Hunter and Wintermantel, 2021), and/or dsRNA ingestion using an artificial feeding system (Yu et al., 2013; Ghosh et al., 2017; Zhu and Palli, 2020). Among these approaches, ingestion of dsRNA in sucrose feeding systems is convenient and affords precise control over ingestion of dsRNA that arrives directly in the gut (Vyas et al., 2017). For this study, the latter approach was implemented to define the following three key features of environmental RNAi for PoP, the minimal effective concentration of dsRNA for effective gene knockdown, persistence of the knockdown, and differential penetrance of RNAi in three psyllid developmental stages, 3rd instar, and teneral and mature adults. Gene knockdown was quantified for each concentration by real time qRT-PCR amplification. Based on the minimal effective concentration established, the persistence of gene silencing was evaluated over a 12-day period.

The PoP targets, *vATPase-A, Snf7*, and *CHC*, exhibited relatively high knockdown at 40%–60%. Because these genes are involved in insect cellular processes their functions are expected to be critical in most tissues and organs during all or most insect developmental stages. The vATPases are involved in metabolic signaling, oxidative phosphorylation, phagosome, endosome, and lysosome formation, the synaptic vesicle cycle and in other pathways that require pH regulation. The *Snf7* and *CHC* genes are involved in endocytosis, and endocytosis and lysosome pathways, respectively. All three genes are conserved across insect orders and have been evaluated in RNAi research for multiple insect species (Baum et al., 2007; Angelotti-Mendonça et al., 2020), making them amenable for comparative analysis of RNAi characteristics with other better-studied insects. Further, optimal RNAi efficacy for biopesticide development rests on the consistent expression of the genes across all insect developmental stages.

For the three candidate genes studied here, the minimal effective dose of the dsRNA was 100 ng/μL (100 parts per million, ppm), which is at least three orders of magnitude higher than the optimal concentration reported for coleopteran environmental RNAi (Baum and Roberts, 2014). These observations underscore the challenges encountered in developing RNAi as a biopesticide for controlling psyllids, and likely for other hemipteran pests. Psyllids have piercing-sucking mouthparts, and they specialize in phloem-feeding. Delivering sufficient, accessible dsRNA into the plant phloem has been challenging for methods such as foliar spray, trunk injection, and laser-etching. Recently, foliar application of dsRNA was shown to be successful when delivered in a matrix of layered double hydroxide (LDH), referred to as BioClay, for whitefly control (Jain et al., 2022). Although this material has not been evaluated for dsRNA delivery in psyllids, the RNAi knockdown in whitefly has been shown to be superior to RNAi effectiveness in psyllids (Mondal et al., 2020; Yyas et al., 2017). Thus, alternative approaches will be required to deliver concentrations of dsRNA sufficient for achieving gene silencing and persistence in the long term, especially for woody plants, such as *Citrus* spp.

The persistence of RNAi-induced gene silencing, a crucial characteristic of RNAi, was investigated, with a mindfulness to applications in the field. This key RNAi feature is not well-studied for psyllids or most other insect or arthropod pests of interest. In general, persistence data are scarce in the literature, the community has not established or implemented consistent methodologies and/or concentration regimes, and at the same time diverse parameters and delivery systems have been adopted across other insect study systems, making comparisons between experimental results difficult. Ingestion-access durations ranging from 2 to 8 days have been used in studies of other hemipterans without knowledge of whether exposure/ingestion times could be too short or too lengthy. Consequently, the interpretation of the results depend on many factors, including accurate functional annotations of gene targets, relevance of the gene to the insect life stage, tissue-specific expression of the gene, and optimal concentration of the dsRNA (Andrade and Hunter, 2017; Galdeano et al., 2017; Ghosh et al., 2018; Kishk et al., 2017; Tanning et al., 2016a; Wuriyanghan and Falk 2013; Wuriyanghan, Rosa, and Falk 2011; Yu and Killiny 2020; Yu et al., 2017). To address at least several of the latter caveats, the persistence of gene silencing at the pre-determined, minimal effective dsRNA concentration of 100 ng/μL (see Results, above) was determined. The RNAi persistence results suggest that in PoP, and potentially in other psyllids, the duration of the dsRNA-induced gene silencing is short-term and persists 4–5 days following removal of the dsRNA source. This indicates that the RNAi triggering molecule, i.e., dsRNA, must be available continuously to perpetuate the silencing effect, which is expected to be essential for achieving a discernible phenotype, such as psyllid mortality or reduced fecundity, among others. Therefore, delivery of dsRNA by viral-induced gene silencing vectors (VIGS vectors) expressing dsRNA hairpins, or transgenic plant-mediated dsRNA expression either from the chloroplast or nuclear genome approaches may represent the most viable options available for dsRNA delivery for psyllid pest and vector control (Hajeri et al., 2014; Dong et al., 2022; He, 2022; Wu et al., 2022).

The low RNAi persistence documented in this study was not unexpected for psyllids. Among invertebrates, RNAi persistent has been shown to be robust in *C. elegans*, and is due primarily to the initially strong systemic RNAi followed by the amplifying effect of the RNAi trigger by the RNA dependent RNA polymerase (RdRP), which further has been shown to propagate gene silencing through several generations (Spracklin et al., 2017). Insects do not encode RdRP and systemic RNAi may not operate in psyllids and/or in other hemipterans. In the pea aphid, the delivery of dsRNA by expression in transgenic plants resulted in knockdown that reached a maximum between day 4 and day 8 (Coleman et al., 2015). Aphids provided longer ingestion exposures to plants did not exhibit increased knockdown, indicating that the RNAi effect reached a plateau after day 8, potentially due to saturation of capacity of RISC. Further, 6 days after the dsRNA source was removed, gene expression levels fell to baseline levels. In another study, expression of pea aphid vATPase subunit E (VAT-E) was silenced by injection-delivered VAT-E dsRNA, and the duration of silencing was diminished after 72 h. This transient RNAi effect was attributed to a rapid degradation of the dsRNA by exo- and endo- nucleases in the pea aphid (Cao et al., 2018). This phenomenon was also observed for psyllids, which also express transcripts of several endonucleases in the gut and salivary glands (data not shown). Also, because the pH of the psyllid gut is basic, dsRNA may not withstand the environment for long periods of time, sufficient to initiate and sustain silencing. The latter barriers potentially limit delivery of any significant amount of dsRNA beyond the gut, resulting in a transient RNAi phenotype. Interestingly, silencing of the *vATPase-A* gene was initiated sooner and persisted longer compared to *CHC* and *Snf7* (Figure 2). Further, a lower dsRNA concentration (10 ng/μL) resulted in significant knockdown of *vATPase-A* expression even though only 20% silencing was achieved at this dose (Figure 1). Together, the results indicate that the dsRNA concentration required to achieve an analogous level of gene silencing is lower for *vATPase-A* than for *CHC* and *Snf7*, or that other biological factors such as systemic properties of RNAi could be responsible for the differences in persistence of their silencing. The higher endogenous expression of the *vATPase-A* gene in psyllid gut compared to the other two genes evaluated (Supplementary Figure S1) might influence knockdown persistence. One possible explanation could be that RNAi more effectively silences genes in the gut epithelium than those expressed in other tissues. Generally, targeting gut-expressed genes results in higher RNAi efficiency in organisms when systemic RNAi is not robust (Vyas et al., 2017). Previously Wuriyanghan et al. (2011), Wuriyanghan and Falk (2013) reported significant knockdown of an ATPase gene in the gut, compared to that for whole body or abdomen samples (Wuriyanghan et al., 2011; Wuriyanghan and Falk, 2013). Although the robustness of systemic RNAi in psyllids is unknown, the greater persistence of knockdown of the *vATPase-A* gene, compared to results for *Snf7* and *CHC,* has demonstrated that gut-localized genes are effectively knocked down by RNAi in potato psyllid.

In *C. elegans*, systemic RNA interference defective 2 (Sid2) protein is responsible for passive transport of the dsRNAs from the intestinal lumen to the epithelial cells, whereas the Sid1 protein carries out passive bidirectional transport of the dsRNA between cells (Tomoyasu et al., 2008; Zhu and Palli, 2020). Though Sid2 orthologs are absent in all insects, there are multiple Sid1-like proteins present in insects including psyllids (Taning et al., 2016b). The red flour beetle *Tribolium castaneum* (Herbst, 1797) has systemic RNAi properties, but Sid1 genes are not required for cell-to-cell transport of the dsRNAs in *T. castaneum;* thus, among different insects the function of these genes are not completely understood (Tomoyasu et al., 2008). The cellular uptake and systemic properties of RNAi appear to be complex and versatile in insects, and there are potentially multiple pathways involved in this process. Thus, the biological mechanisms underlying differences observed in silencing characteristics of PoP *vATPase-A* gene and those of *Snf7* and *CHC* cannot yet be explained.

Finally, the 3rd instar (immature) psyllids were more responsive to environmental RNAi than their counterpart teneral or mature adults. Although RNAi penetrance among the different instars of the same psyllid species, or between species, has not been systematically evaluated, Wuriyanghan and Falk (2013) reported that immature psyllids ingested and accumulated higher levels of viral-derived RNA than PoP teneral adults do (Wuriyanghan and Falk, 2013). Other studies have reported similar trends for the different insect developmental stages. For example, in the nymphal stage of the Sunn pest *Eurygaster integriceps* Puton (Hemiptera: Scutelleridae), gene silencing levels were greater in the nymph than for adults (Amiri and Bandani, 2020). The spotted wing fruit fly *Drosophila suzukii* (Matsumuru, 1937) larval stage also exhibited higher levels of knockdown across multiple genes, where gene silencing was documented at 32%–42% and 19%–24%, respectively, in larval and adult stages, and mortality was 10%–23% in adults, compared to 22%–42% in larvae (Taning et al., 2016b). By comparison, in two coleopteran pests, the southern and western corn rootworms, *v-ATPase-A* and Snf*7* genes showed higher levels of gene silencing in larvae compared to the adults, at 55%–89% and 48%–83% knockdown, respectively. Similarly, gene silencing experiments with the 2nd and 4th early larval stages of the hemipteran *R. prolixus* resulted in a frequency of 42% for the 2nd instar and little or no detection in the 4^th^ instar (Araujo et al., 2006).

The distinctive RNAi responses observed across the potato psyllid life stages are likely attributable to several factors. The overall expression of the three gene targets evaluated here did not differ dramatically between the adult and nymphal stages. However, the feeding habits of the psyllid stages are different, with immatures being more voracious feeders than the adults, which is potentially attributed to physiology of younger instars that experience multiple cycles of rapid growing and molting (George et al., 2017; George et al., 2018). Consequently, the immature stages must be more metabolically active, a scenario that might lead to more rapid processing of ingested dsRNAs, thereby avoiding inactivation or degradation in the basic (high pH) environment of the gut lumen. Whether the pH of the adult gut is more basic than the immatures’ is not known. Also, at least two dsRNA-specific endonucleases are encoded by the psyllids, and transcriptomic evidence indicate these are expressed in the gut and salivary glands. These enzymes are expressed to a greater extent in adults than nymphs (Supplementary Figure S2), which could result in greater or more rapid degradation of dsRNAs ingested by adults than by immature stages. Finally, the RNAi core machinery proteins could possibly be involved in the observed differential expression in psyllid adults and nymphs thereby influencing the differential processing of the exogenous dsRNAs in different or all psyllid life stages. To investigate this possibility, relative expression of psyllid RNAi machinery proteins was calculated from data reported in a previously published study, with manual curation of the datasets (Fisher et al., 2014) (Data Sheet S2, Supplementary Table S1). Interestingly, Dicer2 expression was higher in the nymphal compared to adult instars, while Ago2 expression was similar for the two life stages. These observations may possibility suggest that the nymphs harbored more efficient RNAi machinery; however, the two Dicer2 associated proteins, Loqs and R2D2, could not be identified from the PoP transcriptome, precluding the possibility of a satisfactory explanation for the differential processivity of dsRNAs observed for adults and nymphs. The different levels of RNAi efficiency among the different psyllid life stages may have important ramifications to disease management. If nymphs can be controlled effectively before reaching adulthood, the spread of “*Ca*. Liberibacter” spp. to the plant host could be abated to some extent by reducing the population size prior to reproduction and/or before adult dispersal and colonization of the host plants.

The candidate PoP genes evaluated in this study are conserved among members of the Class: Insecta and their primary biological roles are to carry out fundamental physiological functions. The characteristics of RNAi may vary for genes that encode proteins involved in adaptive or other non-constitutive functions such as those involved in immunity and defenses, or highly specific functions that are essential for one or more life stage. In summary, the RNAi characteristics and parameters established in this study are expected to inform experimental design for RNAi screening and selection of specific phenotypes, e.g., mating interference, mortality, reduced fecundity, and others, by providing baseline results over a range of optimal dsRNA doses to standardize laboratory studies, potentially applicable to PoP and to other psyllid species. The results will also be valuable for the development and optimization of biopesticide studies at the field level, toward effective management of psyllids as pests and vectors of psyllid-transmitted plant pathogens.

Finally, certain environmental and regulatory aspects have been considered in the design of the dsRNA molecules selected for silencing three genes evaluated in this study. The approach involves *a priori* comparisons of regions of the dsRNA with analogous regions of gene orthologs in genome and/or transcriptome databases for beneficial and organisms of interest in different agroecosystems, in the case of PoP, lacewing, lady beetle, springtail, honeybee, and also humans, to avoid predicted gene sequences (threshold of 0–1 identical nucleotides) with potential off-target effects as siRNAs (data not shown) (Alyokhin et al., 2022). Sequence specificity is an advantage of RNAi-biopesticides over most traditional chemical pesticides, which together with rapid degradation of the dsRNAs in the environment, are promising incentives for advancing this new type of technology and its acceptance in integrated pest and vector management programs (Christiaens et al., 2022; Vaschetto, 2022).

## Data Availability

The original contributions presented in the study are included in the article/Supplementary Materials, further inquiries can be directed to the corresponding author.
