# Supplementary material for: Characteristics of environmental RNAi in potato psyllid, Bactericera cockerelli (Sulc) (Hemiptera: Psylloidea: Triozidae)
Source: Front Physiol. 2022 Oct 18;13:931951. doi: 10.3389/fphys.2022.931951 (PMC9623324; doi:10.3389/fphys.2022.931951)
Supplement: Supplementary file 1 [file DataSheet1.docx]

**Supplementary file 1**

**Supplemental figure 1 (A-D):** RPKM (Reads Per Kilobase of transcript, per Million mapped reads) values sequenced from RNAseq libraries of potato psyllid (PoP) teneral adults, 3^rd^-5^th^ instar nymphs, and guts (Ad-Gut) and salivary glands (Ad-SG) extirpated from PoP adults (Ad), for the vacuolar ATPase A subunit (*vATPase-A*) (A), clathrin heavy chain (*CHC*) (B), and non-fermenting protein 7 (*Snf7*) (C) target genes. Ratio of psyllid guts and adult whole body RPKM values for the three target genes are shown in D. Data for this figure was taken and manually curated from Fisher *et al.,* 2014 (Fisher et al., 2014).

**Supplemental figure 2:** Reads Per Kilobase of transcript, per Million mapped reads (RPKM) for adults and nymphs (whole body) obtained by Illumina RNAseq of the dsRNase 1 (BcAN_15309) and dsRNAase 2 (BcAN_05172) libraries. Data for this figure was taken and manually curated from Fisher *et al.,* 2014 (Fisher et al., 2014).

**Primers used for double-stranded RNA synthesis**

vATPase-A F CATCGGGTGTAACTGTTGGTGACCC

vATPase-A R CCATACTGATCTCCACCGGTCATGTG

Snf7 F GGGTTGGATATGGCTTCTGATATCTCCTTGG

Snf7 R GAGAAACAATTGCAACAAATTGATGGCACCC

CHC F TCTGACAAGTTCATCTGCGTGCGAG

CHC R AAGACCACGTCGTCGGTCATGG

Luc F TTCAACGAGTACGACTTCGTGC

Luc R GGTACATCAGCACCACCCGAAAGC

**Real time, quantitative reverse transcriptase-polymerase chain reaction primers and probes**

vATPase-A F CATCCCTGGCTGAATCTGATAA

vATPase-A R GAAGGGACAGAACCTGTCATAG

Probe TGGAAGTTG/ZEN/CCAAACTGCTGAAAGA/3IABkFQ

Snf7 F AAACAGGAGTTTCTGGAGAAGAA

Snf7 R AATGCTTGAATTGCAGCTCTTT

Probe TCAACATAGCTAGAACCAATGGAACCA

CHC F CAGGTGAGCAGCAAGTATGA

CHC R TGCTAATGCGGTTCATGTAGA

Probe TGTACGACATAGAGACGGCCACCT

RPL5 F CTGGTGTGATTGCTGATGATATTG

RPL5 R TCCACAGGTTTGGTGGATTT

Probe AGCTGACCCAACCCATGTCAAGAA

**Potato psyllid v-ATPase A sequence partial coding region**

ATGTCAACAGCATTAGGAAAAATGGTGGATGAAGATAGAGAAGGTAGATTTGGGTTTGTGTATGCAGTATCAGGTCCTGTGGTAACAGCAGAGAAAATGTCAGGGTCTGCTATGTACGAGCTGGTTCGAGTAGGTTACTTTGAACTGGTGGGTGAAATCATCCGGCTGGAGGGTGATATGGCCACCATTCAAGTGTATGAAGAAACATCGGGTGTAACTGTTGGTGACCCTGTGTTGAGGACAGGCAAACCCTTATCTGTAGAGCTTGGTCCTGGTATCCTGGGTAGTATTTTTGATGGTATCCAACGTCCACTGAAGGACATTTGTGAGTTGTCTCAGAGCATTTACATCCCAAAAGGAGTTAACATTCCTGCCTTGAACAGAGATGTTAGCTGGGAGTTCAATCCAATGAACTTAAAGATTGGTAGTCACATGACCGGTGGAGATCAGTATGGTATTGTACATGAGAATACACTTGTTAAACATAAAATGATCATGCCACCCAAAGCAAAGGGAACTGTAACATACATTGCTCCAGCTGGTAATTACAAGGTAGATGAAGTTGTTATTGAAACTGAATTTGATGGAGAGAAGAGTAAATACACTATGGTTCAAGTATGGCCTGTACGTCAACCTCGCCCTGTCACCGAAAAACTCCCTGCAAATTACCCCCTTCTAACAGGTCAACGAGTCCTTGATTCTCTGTTTCCTTGTGTTCTTGGAGGAACAACTGCCATTCCTGGTGCCTTTGGCTGTGGTAAAACTGTCATCTCACAAGCTTTATCTAAGTATTCCAATTCTGATGTGATTGTCTATGTAGGATGTGGTGAACGAGGTAATGAAATGGCAGAAGTATTGAGAGATTTCCCTGAACTTTCAATTGAGGTGGATGGAGTCACAGAATCCATCATGAAACGTACCACCTTGGTAGCCAACACATCAAACATGCCTGTAGCTGCTCGAGAAGCTTCTATTTACACTGGTATCACACTGTCTGAGTACTTCAGGGACATGGGTTACAATGTGTCTATGATGGCTGACTCCACGTCACGATGGGCTGAGGCTTTGAGAGAAATCTCAGGACGTCTAGCTGAGATGCCTGCTGACAGTGGGTATCCTGCTTACCTGGGTGCAAGACTTGCCTCCTTCTATGAGCGTGCTGGTAGAGTTAAGTGTTTGGGTAACCCAGACAGAGAGGGTTCTGTTAGTATTGTGGGTGCTGTGTCTCCCCCTGGTGGTGATTTCTCAGACCCTGTCACTTCAGCAACATTGGGTATTGTTCAAGTGTTCTGGGGTCTTGACAAAAAACTTGCTCAGAGGAAACATTTTCCATCCATCAACTGGCTCATTTCCTACAGTAAGTACATGAGAGCCCTGGATGATTTCTATGAAAAAAACCACCCTGAGTTTGTACCCCTCAGAACAAAAGTAAAAGAAATTCTTCAAGAAGAAGAAGATTTGTCAGAAATTGTGCAACTGGTTGGTAAGGCATCCCTGGCTGAATCTGATAAGATCACTTTGGAAGTTGCCAAACTGCTGAAAGATGATTTCCTCCAACAAAACAGTTACTCCCCCTATGACAGGTTCTGTCCCTTCTACAAAACTGTTGGTATGTTGCGTAATACTATTGCTTTCTATGATATGTCCCGTCATGCAGTTGAGTCTACTGCCCAATCAGAAAACAAGATCACATGGTCTGTGATAAGGGACAGTATGAACAACATCTTGTATCAACTTTCGTCCATGAAATTCAAAGACCCAGTCAAGGATGGTGAAGCTAAAATAAGAGCAGACTTTGATCAACTCTATGAAGACATTCAGCAAGCATTCCGTAACTTAGAAGACTAA

**Potato psyllid Snf7 sequence partial coding region**

ATGAGTTTCTTCAAAAACATCTTCGGGAAAACTGAGGAAAAGGGCCCCACCACAGGAGAGGCCATCCAAAAACTGAGAGAAACAGAAGACATGCTTATAAAGAAACAGGAGTTTCTGGAGAAGAAAATAGAACAGGAAATCAACATAGCTAGAACCAATGGAACCAAAAACAAAAGAGCTGCAATTCAAGCATTGAAAAGAAAGAAAAGGTATGAGAAACAATTGCAACAAATTGATGGCACCCTGTCAACTATTGAGATGCAACGGGAAGCTCTGGAGGGTGCTAACACAAACACTGCGGTACTTACCACCATGAAGAATGCAGCTGATGCACTCAAGGCAGCACATAAACACATGGATGTGAACCAGGTGCATGATATGATGGATGACATTGCGGAACAGCAAGACGTAGCCAAGGAGATATCAGAAGCCATATCCAACCCTGTGGCCTTTGGACAAGATGTGGATGAGGACGAGTTGGAGAAGGAGCTGGAGGCCCTGGAACAGGAAGAGCTGGACAAGGATCTCCTGAAGCTGAGTACTCCCGGGGGAGAGTTGCCAGAGATCCCCACCACGGCGCCCAAGGAGAGACCAAAGGAGAAAGCCAGCACCAAGGACCGCTCTGTGGAGGACGAGATCAAGGAATTGGAAGCGNTGGGCTTCGTAATATATATCCGACCCAGCCCACCCCGGACCCAGCCATCCCATGAATTGTCTAACAATTGCTTGCGTAAGCTAAGGCTCGAGCTATATGCCTACCCACTTGTATGCGTAGGCTCGCCAGGTCACACATTTACGTAA

**Potato psyllid CHC sequence partial coding region**

ATGACACAACCATTGCCCATCAAGTTCCAGGAACTACTCCAGCTCACCAGTGTGGGCATCAGTCTGGGGAGCATCAGCTTCAACACCCTCACCATGGAGTCTGACAAGTTCATCTGCGTGCGAGAGAAGATCGCGGATTCCGCGCAGGTGGTCATCATCGACATGAACGACCCCACCACCCCCATCCGGAGACCCATCAGTGCCGACTCCGCCATCATGAACCCAGCCAGCAAGGTGATAGCTCTCAAGGGCAAGGCTGGCAACGACAATAATCCCAACGCGCCCAAGACGCTGCAGATCTTCAACATCGAGATGAAGTCGAAGATGAAGGCTCACCCCATGACCGACGACGTGGTCTTCTGGAAGTGGATCTCGCCCAACACCCTGGCCCTCGTGACGGAGACCTCGGTGTACCACTGGAGCATGGAGGGCGACTCCCAGCCGGAGAAAATGTTCGACCGCCACTCCACGCTGAACGGGTGTCAGATCATCAACTACCGCACAGACCCAAAGCAGACCTGGCTCCTCCTGGTGGGAATCAGTGCCGTTCAGAACAGAGTGGTGGGGGCGATGCAGCTCTACTCTGTGGAGCGCAAGTGCTCCCAGCCTATTGAAGGCCACGCCGCCTCGTTCGCCACGTTCAAGTCCGAGGGGAACCCGGAGCCCGCCACTCTGTTCTGCTTCGCGGTGAGAACGGCGGCGGGCGGCAAGCTGCACATCATAGAGGTGGGCACCCCGCCCGCCGGCAATACGCCGCTGGCGAAGAAGGCGGTGGACGTGTTCTTTCCGCCCGAAGCCGCCACTGACTTCCCGGTGGCGATGCAGGTGAGCAGCAAGTATGACGTCATCTACCTGATCACTAAGTACGGCTACATCCACGTGTACGACATAGAGACGGCCACCTGCATCTACATGAACCGCATTAGCAGCGATACGATCTTCGTGACGGCGCCCCACGAGGCGACGGGCGGCATTCTGGGCGTCAACCGGAAGGGCCAGGTGCTCTCGGTGAGCGTGGACGAGGACAATATCATCCCCTACATCAACGGCACCCTACAGAACCCTGACCTGGCCCTGCGGATAGCTGTGAGGAACAATCTGGCCGGCGCTGAGGACCTGTTCGTACGCAAGTTCAATCTTCTCTTCACCAACGGGCAGTACTCTGAGGCCGCCAAGGTGGCAGCCAACGCGCCCAAGGGCATCCTGCGCACCCCCCAGACCATAGTCAAGTTCCAGCAGGTGCCCACCCCTGCGGGCCAGAACTCCCCCCTCCTCCAGTACTTCGGTATCCTGCTCGACCATGGCAAGCTCAACAAGCACGAGAGTCTGGAGCTGTGTCGCCCAGTGCTGGCCCAGGGCCGGAAGCATCTGCTGGAGAAGTGGCTCAAGGAGGAGAAGCTGGAGTGCTCGGAGGAGCTGGGGGATCTGGTGAAGCAGTCGGATCCGACCCTGGCTCTGAGTGTGTACCTGCGAGCTAATGTTCCTAACAAGGTGATTCAAAGCTTCGCCGAGACGGGCCAGTTCCAGAAGATCGTGCTGTACGCCAAGAAGGTGGGCTACACCCCCGACTACGTGTTCCTGCTGAGGAATGTGATGCGGGTCAATCCCGACCAGGGGGTGGGCTTTGCGCAGATGCTGGTGCAGGATGACGAGCCCCTGGCAGACATAAACCAGATCGTGGACATCTTCATGGAGCAGAACATGGTGCAGCAGTGCACCGCCTTCCTGTTGGACGCCCTCAAGAACAACCGGCCCGGCGAGGGGCCGCTCCAGACCCGCCTCCTCGAGATGAACCTCATCTCCGCCCCCCAGGTGGCCGATGCCATACTGGGCAACCAGATGTTCACGCACTATGACCGCGCCCACATTGCCGCCCTGTGCGAGAAGGCGGGGCTTCTGCAGCGCGCCCTGGAGCACTACACAGACCTGTACGACATCAAGCGGGCCGTGGTGCACACTCAGCTCCTGAACGCGGACTGGCTGGTGGGCTACTTCGGTACCCTCTCCGTGGAGGACTCGCTGGAGTGTCTCAAGGCCATGCTCACCAACAACATCCGCGCCAACCTGCAGATCTGCATCCAGATCGCCACCAAGTACCACGAGCAGTTGACCACCAAGGCGCTGATAGATCTGTTCGAGTCTTTCAAGAGCTACGAGGGTCTCTTCTACTTCCTCGGGTCCATAGTCAACTACAGCCAGGACCAGGAGGTTCACTTCAAGTACATCCAGTCGGCCTGCAAGACAGGCCAGATCAAGGAGGTGGAGCGCATCTGCCGCGAGTCCAACTGCTACAACGCGGAGCGCGTCAAGAACTTCCTCAAGGAGGCGAAGCTCACCGACCAGCTGCCGCTCATCATCGTGTGTGATCGGTTCGACTTCGTCCACGACCTCGTCCTCTATCTGTACCGGAACAACCTGCAGAAGTACATCGAGATCTACGTGCAGAAGGTGAATCCCTCTCGTCTACCAGTGGTGGTGGGGGGACTACTGGATGTGGACTGTAGCGAGGACATCATCAAGAATCTGATCCTGGTGGTGAGAGGACAGTTCTCCACCGATGAGCTGGTAGAAGAGGTGGAGAAGCGCAACCGTCTGAAGCTCCTGCTCCCCTGGCTGGAGTCTCGCATACACGAGGCCTGCACCGAGCCCGCCACTCACAACGCGCTCGCCAAGATCTACATCGACAGCAACAATAATCCCGAGCGGTTCCTCAAAGAGAACCAGTTCTACGACAGCCGTGTGGTGGGGAAGTACTGTGAGAAGCGGGACCCCCACCTAGCCTGCGTGGCCTACGAGAGAGGAGCCTGCGACCGGGAACTCATTCAGGTGTGCAACGAGAACTCCCTGTTCAAGTCCGAGGCCCGCTACCTGGTGAGACGCAAGGACCCCGAGCTCTGGCTGGAGGTGCTCTCCGAGGCTAACCCGTTCAAGCGACAGCTCATCGATCAGGTGGTCCAGACCGCCCTCTCAGAGACTCAGGACCCAGAGGATATCTCCGTCACAGTCAAGGCCTTCATGACGGCGGATCTGCCCAATGAACTCATCGAGCTGCTGGAGAAGATAGTGCTGGACAACAATGTGTTCAGCGATCACAGGAACCTCCAGAACCTCCTGATCCTGACCGCCATCAAGGCGGACCAGACGCGCGTGATGGAGTACATCACCCGCCTGGACAACTACGACGCCCCCGACATAGCCAACATCGCCATCAACAACCAGCTGTACGAGGAGGCGTTCGCCATCTTCAAGAAGTTCGACGTGAACACGAGCGCCATCCAGGTGCTGATCGAGAATGTGGGCAATTTGGACCGGGCGTACGAGTTTGCTGAGAGATGCAACGAGCCGGGCGTGTGGTCTCAGCTGGCCAAGGCCCAACTAGCGCAGGGCCTGGTCAAGGAGTCCATCGACTCTTTCATCAAGGCGGACGACCCGAGCGCCTACCTAGACGTGGTGGCGACGGCGCACCAGACCGAGTCCTGGGAGGACCTGGTTCGATACCTCTCGATGGCGCGGAAGAAGGCGCGCGAGAGTTATATTGAGAGTGAGCTGATCTATGCGTACGCGCGCACCAACCGGCTGGCAGACCTGGAGGAGTTCATCTCAGGCCCGAACCACGCCGACATCCAGAAGATCGGCGACCGCTGCTTCGAGGACCGCATGTTCGAGCCCGCCAAGATTCTCTACAACAACGTGTCCAACTTCGCCCGCCTCGCTCTCACCCTCGTGCATCTGAGGGAGTACCAGGGCGCCGTGGACTCGGCGCGCAAGGCGAACAGCACACGCACCTGGAAGGAGGTGTGCTTCGCGTGTGTGGACTCCGGGGAGTTCCGACTGGCGCAGATGTGCGGCCTCCACATAGTGGTGCACGCGGACGAGCTGGAGGACCTGATCAACTACTACCAGGACCGCGGCCACTTCGAGGAGCTGATCAACCTGCTGGAGGCCGCGCTGGGGCTGGAGAGGGCCCACATGGGGATGTTCACCGAGCTGGCCATCCTCTACTCCAAGTACAAGCCCGCCAAGATGAAGGAGCACCTCGAGCTGTTCTGGTCCCGCGTCAACATTCCCAAGGTGCTGAGAGCAGCAGAGCAGGCACACCTCTGGTCAGAGCTCGTGTTCCTGTATGATAAGTACGAGGAGTACGACAATGCCGTCATCGCCATGATGACTCATCCGACTGAGGCATGGCGGGAGAGTCATTTCAAGGATATCATCACCAAGGTTGCCAACATTGAGCTCTACTACCGCGCCATCCAGTTTTACCTGGACTACAAGCCTCTGCTCCTGAACGACATGCTGCTCGTTCTCACACCCCGTATGGACCACACGCGCTCGGTAGCCTTCTTCGCCAAGAGCGACAACCTCCAGCTCGTGAAGCCCTACCTGCGCTCCGTCCAGTGTCTCAACAACAAGGCGATCAACGAGGCGCTGAACCAGCTGCTCATAGACGAGGAAGACTACCAGGGTCTGCGCACAAGCATCGACGCGTTCGACAACTTCGACACGATCGCGCTTGCGCAGAAACTGGAGAAGCACGAGCTGACNNNNAAGAAGGACAGACTGTTCAAGGACGCGATGGAGTACGCGGCCGAGTCCAAGAACACAGAGCTGGCCGAGGAGCTGCTCGCCTGGTTCCTGGAGCGTCGCAACTTGGACTGCTTCAGCGCCACCCTGTACCAGTGCTACGACCTGCTGCGGCCTGACGTCATCCT

**Potato psyllid dsRNase 1 (BcAN_15309) sequence partial coding region**

ATTTTGAAAGAACCTCAAGACTTTGCTAAAAATCACTCCGTCTTTGAAACTTCAGAATTTGTTTACTGAAACTTCAGAATGCCCTTGATATCCGGCACAGGTACAAATGAAACATTGGTGCTCATCTCACGAGGATCACAACAGTAGCAAAATCCTTTCTTGTAGTCGTCTTGCAGTTCTGACCAGTTGTTGCTCGCACAAATGTCAGTTTTACAAAGCAACTCAGGAGGGTTCTCCAGGAAAGGGTCGTTGCTACACACCAGGGCTATAGCCAGGCTGGATTGAGGCTCGTATATGATTTTGAAGAACTCCGTTGGAACTGGTATGCCCTTGTTTCTAGGATCCAAGTACACGGGTGTGTTTGCAAGGGTCAGTATTCCGTGAGTCCCGGTGGTTACGTCCAGAGTTGTGCCAATATTCTCCGCCAGCTGTCGCACAGCCCTCTCCACCTTGAGCCAGTTGCCGGCGTTGATGCTCTGGAACTGGGGGCTCACGTTGGCGTAATAGTAGGTGGCCCACTGCCAGGCCGCCATCAGGAAGTCCCCATCAGGAGCCAGGTGGCCTCGGGACATGAACATCTTGCTGTTGATGTACTTCTGGGCCTCGGCCTTTGAACCGAGGATATTTTCGAAAACGCGAACCTGTTGTGCCAAGTTATAAGCCTTGTTCGGTGGGAGCCCTTTGAACATTGATTTGTCTCCCACTCGGAAGTTGGGTCTCTTGCTCTGGCTCTGAGCACCCTGTATGCTGGGGAAGACCTGGTGTCGGGTGTACAGGGTGCGGGTCTCCTTCACGTTGTGGCACAACGAGAACAAAGTAGCCAAAGTATCGCCCTCCACCTGGAAGCCAATATTGAAGATCAATGCATTTTCCGAACCACCACATTTTGTTTTTGTGGCTTTGATGTATGAATCAGGGTTTTTCCTACACACCAAATTATCTTCAGATAAGACATCGCCGTCTGCGTTCACATTTCCGTTATTTCCACATTTTCCTGTGACCTTGCTCTCCTCGAACTCCTCGAGTGAATTTCCTTTTCCAGGACACAAGAGAATGACCTCCTGGTTTTTCTGTAAAGAGAGGGCCCCATGGTTTTTTTTTCNNTGGTTTGTCTGCAAAGAGAGAGCCCCATGGTTTTTTTTCACTGTGAGTTCAGGAATAATCAAATTGGAGTTACTATCAACGAAAACTGGCTGATTATCAAACGGAATGTCGTCCTGCAGATCCAGTTGGCATTCTTGTGAATAAGCAGAATTAAAGAACACTATTGAAAAGAAAATAACGGTTGAACAACGAAAAAACAAACATCTTTGATTGGAATACATTTAAGGAAGTGGTTGTTGTAGCTCGTGGATTTGGTATGACAAAAAAGAAATGAAGCACTCAGGAATTCAAGCTATAAAAACAGAAGAAGAAAAAAATCACAT

**Potato psyllid dsRNase 2 (BcAN_05172)sequence partial coding region**

TTCATCTTCTATAGGCCAGAAAGTGTGCGATGTGATCCAACTACTTTAGTCTTGTCGGAAGATATCCTGACAACGGTCAGTCTGTCGGGTTATTCAGTCGGTATTAATAGGTTTTGATTAGTGATTGAGGAACCGAGAAACATGGTTCAATATTGGGTCGTTTTAGCGACATATTTGGTCTCTTTTCATTTCAAAGAAGTGTTTTGCCGAACCACATGTAGCATTAATTTGAACACGGAATATCGCGCGTCAGAGGAGCCTTTGTTTTTGAGTCCTGACGGTGCGAGTATCGTCTACCCTAACAACAGAGCCAGCATTGAAGTCCACGGAGGAGACAAACTCAGAGTTTACTGTGGACACAAGAACTTCAAGGAGCTGAAATCTCACTCGGACGACTCCCTCTTGGTTCAATGCAACAGTGGGAAATCCTTCAAGCTCCTCTCTACTCATCTGCACAATGCCCAGTCTGTCCAATATTCTCAATCTGACAAGGACGTACCTTCCGACTTCTCAACTGGATCCTCAGATCTCCGTCGAACTCCGCACTCCAGCGCTTCTCTAGAACCAAAATCTTTCGCCTGCTCGTCTATTGTTCGCTCTGCAATACGGCCTGGCAAGCCATGTGGGTTGAGGGGTAGAGAGGAGCATCATCAAGAGTTCAGCATAGGATTCCCGATAAGCAAAGGATTTCTTCCCATTATATCAGTATGTTTCAATACCGAACTGAAATCGGTTCTCTGGTCCAGGCATATCGTGTCACCTACAGTGGAATATCACATGAGCAAAGTACCGCGCATTACATTCATTCAGGATAGAATCCCTTACGATGGATCTCACCTCAACGTTGATAAGCTTTACAATCGACATCAAGAAATAGAAACAATCGCAGCTATTCTCGGTTCCAAGGATCTAGCTGAGAAATATATTGAACCCAAGGGGAACGGATTCTTTGCGAGGGGACATTTGGCACCCAAGGGGGACTTTGTGTATGCTGGGGAACAGCTGGCTACCTTCCACTTTATCAATGTGGCACCCCAGTGGCAGTACTTCAATGGAGGACAGTGGGAGAAGATCGAGTCTACTCTCCGCAACTACGTCATCAGGACCAGGGTGCAAGCCAGGGTACTCACTCTTAGCCTCGGCATAACGCAGCTACAGGACGAGGAGGGGGAGGACAGGGACATCTACCTCTACTATAACCAGAGGACGACGGACAAACTGATCAAAGTTCCCAAACTCTACGCCAAGATTATCAACTTTGGCAACAGCTCAGCATCATCCAAGAGTGTGGTCATAGTGGGGGTGAATCAGCCCTATGAGAGTCATGTGAAAATGTCAACCAATGATATCATCTGCCCGGACGTTTGCGACAGGTACGAATGGATAGCGGGTGTCTCCCAGCACAATGACCAGCATCATAGTTCCGGCTACATCTACTGCTGCACTCCCTCAGACTTCATCTCCTACCTACAGTCAGGACAAGGCTACAAGCTCGACTCAGCTGCAGCCCCAGGCAACTCTGACTCACATGAAAAAGCACTAGATTATAATGCTAAAGCTGAATCACATGGTATTCGTTTTCGATTCAGTGAAAATGTTACTTTCCAAGATGGTTTTGGATTGAATGAGCCTGCTGATTCGAATAATCGTTTTGGATTAAATGAAACACCCCTGTCGTATGGGGATCGTGAATACAACAAAAATACGGATTCTCTTGCAGATAGTTCAAGTTTGGATGATTTCAGTAACAGTGCTCTGCAGGTCACTGAGGAAGCTGCCGACACGCTCATAGGACACCTGAGTGACTTTTTAGGTGCTACCTTCTCATATAGATAATTATTACGGCAATGATATTAATTATGACAGAAAACATGAGATATTTCTTCAAAGTAGTTTTGAGTCTATCTCTGAGGTAAACAGGGTGGTGCGGCAACTTTGAAAAATTAATAAAATTGCGAGTTTCATAGATTAAAGAAAAAATATTTTTGTAATGGACCGAG

**References**

Fisher, T., Vyas, M., He, R., Nelson, W., Cicero, J., Willer, M., Kim, R., Kramer, R., May, G., Crow, J., Soderlund, C., Gang, D., Brown, J., 2014. Comparison of Potato and Asian Citrus Psyllid Adult and Nymph Transcriptomes Identified Vector Transcripts with Potential Involvement in Circulative, Propagative Liberibacter Transmission. Pathogens 3, 875–907. https://doi.org/10.3390/pathogens3040875
